# The Microecological-Immune Axis in Pediatric Allergic Diseases: Imbalance Mechanisms and Regulatory Interventions

**DOI:** 10.3390/nu17182925

**Published:** 2025-09-11

**Authors:** Ziyi Jiang, Jie Zhu, Zhicheng Shen, Linglin Gao, Zihan Chen, Li Zhang, Qiang Wang

**Affiliations:** Institute of Infection, Immunology and Tumor Microenvironment, Hubei Province Key Laboratory of Occupational Hazard Identification and Control, Medical College, Wuhan University of Science and Technology, Wuhan 430065, China; 18694017286@163.com (Z.J.); 17703698702@163.com (J.Z.);

**Keywords:** allergic diseases, children, microbiota-immune crosstalk, microbiota modulators, skin–lung–gut axis

## Abstract

In recent years, the global prevalence of pediatric allergic diseases—including atopic dermatitis, allergic rhinitis, and asthma—has increased significantly. Accumulating evidence underscores the pivotal role of the microbiota–immune axis in the regulation of immune tolerance, wherein microbial dysbiosis is a critical driver in the onset and progression of these conditions. Notably, reduced microbial diversity and imbalanced proportions can also cause immune dysregulation and cross-organ signaling. The skin–lung–gut axis has emerged as a key conduit for multi-organ immune communication. Microbial communities at barrier sites not only mediate local immune homeostasis but also influence distant organs through metabolite production and immune signaling pathways, forming a complex network of organ crosstalk. This mechanism is integral to the maintenance of both innate (e.g., epithelial barrier integrity and phagocytic activity) and adaptive (e.g., the Type 1/Type 2 cytokine balance and regulatory T cell function) immunity, thereby suppressing allergic inflammation. Early microbial colonization is crucial for immune system maturation, and its perturbation is strongly linked to abnormal allergic immune responses. As such, the skin–lung–gut axis functions as a cross-organ microecological–immune regulatory network that is particularly relevant in the context of infantile allergic disorders. Intervention strategies targeting the microbiota—including probiotics, prebiotics, synbiotics, and postbiotics—have demonstrated potential in modulating host immunity. Furthermore, emerging approaches such as engineered probiotics, advanced delivery systems, and fecal microbiota transplantation (FMT) offer promising therapeutic avenues. This review provides a comprehensive overview of microbiota development in early life, its association with allergic disease pathogenesis, and the current progress in microbiota-targeted interventions, offering a theoretical foundation for individualized prevention and treatment strategies.

## 1. Introduction

**Figure 1:**
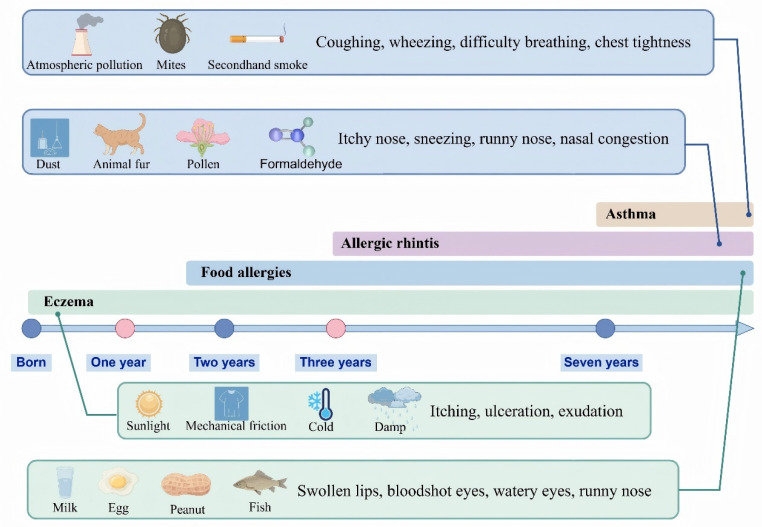
The allergy march and its main causes. The concept of the “allergic march” refers to the progressive sequence of allergic conditions that typically begins with eczema in infancy and may evolve into asthma and allergic rhinitis during childhood. Each of these conditions is influenced by distinct etiological factors. This figure was drawn using Figdraw software (https://www.figdraw.com/).

In recent years, the prevalence of allergic diseases has risen sharply, emerging as a major global public health concern. According to the World Allergy Organization, approximately 30–40% of the global population is affected, with particularly high incidence rates observed among children in industrialized countries [1]. These disorders not only compromise quality of life but can also be life-threatening in severe cases. Common allergic diseases—including atopic dermatitis, allergic asthma, and allergic rhinitis—are characterized by exaggerated immune responses to otherwise innocuous environmental antigens [2]. Clinically, these diseases frequently exhibit a sequential progression known as the “allergic march,” typically beginning with atopic dermatitis during infancy, followed by asthma and allergic rhinitis in later childhood (Figure 1) [3]. Immunologically, allergic reactions are categorized as immunoglobulin E (IgE)-mediated, non-IgE-mediated, and of mixed types [4]. In IgE-mediated hypersensitivity, allergen-specific IgE antibodies bind to high-affinity Fc epsilon receptor I (FcεRI) molecules on the surface of mast cells and basophils. Upon subsequent allergen exposure, the cross-linking of these surface-bound IgE-FcεRI complexes triggers cell activation and degranulation, initiating a cascade of inflammatory events [5,6]. Eosinophils are subsequently recruited and contribute to tissue damage through the release of cytotoxic granule proteins, while Type 2 immune cells amplify the response by producing cytokines such as interleukin-5 (IL-5) and IL-9, thereby establishing a self-reinforcing positive feedback loop [7]. Infants are particularly susceptible to allergic diseases due to the immaturity of their immune systems. During fetal development, Type 2 immune responses dominate. After birth, exposure to environmental microorganisms promotes the differentiation of Type 1 responses, thereby contributing to the reestablishment of the Type 1 cytokine/Type 2 cytokine balance, which is essential for immune homeostasis [8]. However, modern lifestyle factors—such as the overuse of antibiotics, hyper-sanitized living environments, and increased urbanization—significantly limit early-life exposure to diverse and immunomodulatory microbial communities, particularly those associated with natural or agricultural environments. Successive iterations of the “hygiene hypothesis” have expanded our understanding of how insufficient microbial exposure in early life leads not only to the impaired development of Type 1 responses but also to more profound disruptions in immunometabolic programming and the immune regulatory circuits [9,10]. Consequently, microbiota dysbiosis has been increasingly recognized as a key contributing factor in the pathogenesis of allergic diseases.

Recent research has highlighted the intricate interplay between the microbiota and the immune system across the skin, lungs, and gut, which are collectively referred to as the skin–lung–gut axis. This axis facilitates cross-organ immune regulation through both microbial metabolites, such as short-chain fatty acids (SCFAs), and immunoregulatory mediators, including IL-10 and transforming growth factor-β (TGF-β). Local microbial communities not only shape immune responses at their respective sites but also exert systemic effects. For example, gut microbiota modulate pulmonary inflammation via the gut–lung axis, whereas links between gut and skin microbiota point to a shared pathogenic mechanism in atopic dermatitis [11].

These insights have positioned microbiota-targeted therapies as a rapidly expanding area of investigation. Conventional strategies—including probiotics, prebiotics, synbiotics, and postbiotics—aim to restore microbial homeostasis by strengthening mucosal barriers, regulating host metabolism, and suppressing pathogenic organisms. In parallel, emerging approaches such as FMT, engineered probiotic strains, and advanced cell–probiotic delivery systems have shown promise for re-establishing immune equilibrium. Through the multi-level modulation of host–microbe interactions, these interventions represent novel and potentially transformative strategies for the management of allergic diseases associated with microbial dysbiosis. This review provides a comprehensive summary of early-life microbial colonization along the skin–lung–gut axis, its critical role in immune maturation, and recent advances in microbiota-based preventive and therapeutic approaches for allergic diseases.

## 2. Gut Microbiota Development and the Risk of Allergic Diseases

### 2.1. Prenatal Period

Recent studies have illuminated the early colonization of the gut microbiota, thereby challenging the long-standing “sterile womb” hypothesis, which posits that fetuses are devoid of microbes until birth [12]. Contrary to this theory, a microbial presence has been detected in amniotic fluid, umbilical cord blood, placenta, and meconium, suggesting that maternal gut microbiota may traverse the placenta during pregnancy and play a role in the early development of the fetal gut microbiota [13]. This hypothesis is further supported by epidemiological evidence, which demonstrates that children raised on farms—particularly those whose mothers were exposed to barn environments during pregnancy—exhibit lower rates of allergic diseases. For example, prenatal exposure to horse stables significantly reduces the risk of atopic sensitization in infants (odds ratio (OR) 0.58; 95% confidence interval (CI), 0.39–0.86), whereas postnatal exposure shows no significant effect (OR 0.96; 95% CI, 0.63–1.46) [14]. Further investigations reveal that maternal environmental exposure influences the expression of immune-related genes such as TLR-2, TLR-4, and CD14 in offspring, suggesting that environmental factors may influence innate immune development as early as the intrauterine stage [15]. In addition to these findings, perinatal microbiota and their metabolites—particularly SCFAs and exosomal miRNAs derived from breast milk—play a crucial role in regulating allergic susceptibility through epigenetic reprogramming [16]. SCFAs, especially butyrate, significantly promote histone acetylation (H3K9/K27ac) at the *FOXP3* gene locus by inhibiting histone deacetylases (HDAC9/6), thereby opening chromatin structures and facilitating regulatory T cell (Treg) differentiation. Simultaneously, SCFAs activate the downstream signaling pathways (e.g., MAPK/mTOR) via G protein-coupled receptors (GPR43/FFAR2), synergistically enhancing *FOXP3* transcription factor expression to maintain immune tolerance [17,18]. Notably, while these mechanisms are similar in both mice and humans, human immune regulation is characterized by more complex metabolic dependencies, multidimensional epigenetic modifications (including acetylation, demethylation, and acylation), and distinct protein stabilization mechanisms [19].

### 2.2. Mode of Delivery

**Figure 2:**
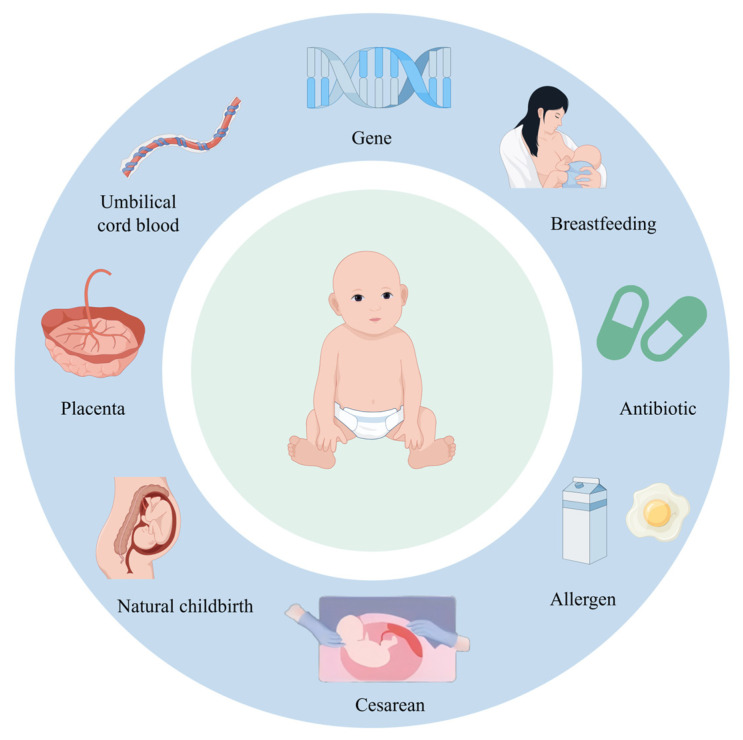
Factors affecting the gut microbiota of infants and young children. Multiple factors during delivery, as well as throughout the prenatal and postnatal periods, can significantly influence the composition and development of the intestinal microbiota in fetuses and infants. This figure was drawn using Figdraw software (https://www.figdraw.com/).

The delivery process significantly influences the microbial composition found in infants, with different delivery modes leading to distinct infant microbiota [20] (Figure 2). The primary sources of the infant’s microbiota include the microbial communities present in the mother’s vagina, intestines, and skin [21], as well as the environmental microbiota encountered by the newborn. Infants born through vaginal delivery predominantly acquire their microbiota from the mother’s vagina and intestines [22], with their gut hosting abundant species such as *Bacteroides*, *Bifidobacterium*, and *Lactobacillus* [23]. In contrast, infants delivered by cesarean section mainly acquire their microbiota from the mother’s skin and the hospital environment, often including species such as *Staphylococcus*, *Streptococcus*, and *Clostridium* [24,25]. Compared to vaginally delivered infants, those born via cesarean section experience the delayed colonization of *Bifidobacterium* species in the gut [26], which can affect the maturation of their immune system and increase susceptibility to conditions like diarrhea, food allergies, and asthma. Furthermore, Nicole Charlotte Steiner and colleagues have demonstrated that *Lactobacillus* species play a crucial role in maintaining the balance between Type 1 and Type 2 immunity, reducing the levels of pro-inflammatory cytokines (such as IL-6), and inhibiting excessive IgE secretion [27].

### 2.3. After Birth

Multiple factors shape the development of the infant gut microbiota, including feeding practices, medication exposure, and the timing of introducing allergenic foods. Among these, feeding practices exert a particularly direct influence on early microbial colonization and the maturation of the immune system [28,29]. Exclusive breastfeeding provides infants with human milk oligosaccharides (HMOs), probiotics, and immunologically active components, which together promote microbial diversity, reinforce intestinal barrier function, and facilitate the establishment of immune tolerance [30]. Despite these benefits, the rate of exclusive breastfeeding in China during the first six months of life remains only 29%, notably lower than the global average of 43% [31]. This suboptimal rate may contribute to an increased risk of immune dysregulation in the infant population. Exposure to medications during infancy—especially antibiotics, proton pump inhibitors (PPIs), and histamine-2 receptor antagonists (H2RAs)—also significantly alters gut microbiota composition. Among these, PPIs have been shown to markedly increase both the prevalence and abundance of disease-associated microbial species, including *Fusobacterium nucleatum* and *Streptococcus anginosus* [32,33,34]. These changes are commonly accompanied by reduced microbial diversity, a decline in beneficial microbial taxa, diminished production of SCFAs [35], and increased intestinal permeability. Collectively, such disruptions can impair immune system development and elevate the risk of infections, metabolic disturbances, and allergic diseases [36]. The timing of allergenic food introduction is another crucial determinant of immune outcomes. Introducing allergenic foods—such as peanuts—within the immunological “tolerance window” (typically between 4 and 6 months of age) has been shown to reduce the risk of food allergies [37]. Conversely, the introduction of milk or egg proteins either too early or too late is associated with elevated allergen-specific IgE levels, indicating increased sensitization and a higher allergy risk.

## 3. Skin–Lung–Gut Axis Microbiota

### 3.1. Interaction Between the Skin Microbiota and Lung Microbiota

As microbiota research advances, accumulating evidence suggests that the skin and lungs—previously considered distinct barrier systems—interact through their respective microbial communities. Under physiological conditions, the skin maintains its microbiota through the stratum corneum and sebum [38], while the lungs rely on mucus secretion, coordinated ciliary activity, and immune surveillance to protect against pathogens [39]. Although their microbial ecosystems are largely independent, studies have demonstrated that skin-associated microbiota, such as *Staphylococcus aureus* and *Propionibacterium acnes*, can transiently colonize the upper respiratory tract via airborne transmission or hand-to-face contact [40], potentially disturbing local immune homeostasis. Conversely, respiratory dysbiosis—characterized by an over-representation of oral or nasal pathogens—may precipitate localized infections if the skin barrier is breached. For instance, one reported case involved an elderly diabetic patient who developed cellulitis and abscesses due to a foot wound infected by *Eikenella corrodens*, a microbiota that is commonly found in the oral cavity [41].

Two primary mechanisms are thought to underpin this cross-site microbial interaction. First, respiratory infections such as influenza or respiratory syncytial virus (RSV) can compromise mucosal defenses, facilitating the migration of skin-derived microbes. Second, chronic dermatological conditions like atopic dermatitis can stimulate the release of systemic immune mediators—such as IL-33 and IL-36 [42]—which, in turn, modulate pulmonary immunity and promote airway inflammation. Supporting this systemic connection, animal studies have shown that cutaneous infections can elevate IgE levels and induce allergic airway inflammation, underscoring the role of immune crosstalk between skin and lung compartments.

**Figure 3:**
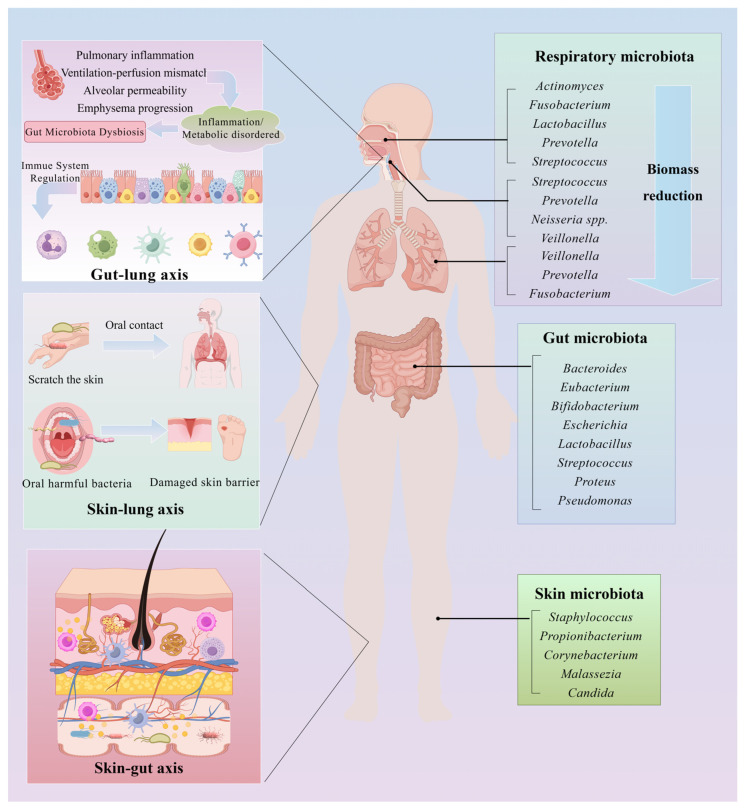
Microbiota and immune crosstalk in the skin–lung–gut axis. The composition of the human skin, lung, and intestinal microbiota varies, and these microbiota interact with one another, facilitating immune crosstalk across different organ systems. This figure was drawn using Figdraw software (https://www.figdraw.com/).

This evolving understanding has prompted the development of innovative strategies aimed at modulating the skin or lung microbiota to reduce systemic inflammation. Such approaches include reinforcing the skin barrier with topical probiotics, stabilizing the pulmonary microbiota through inhaled microbial formulations, and implementing adjunctive measures such as ceramide-based barrier repairs or enhanced oral hygiene to minimize microbial transmission [43]. Clinically, these insights offer promising therapeutic avenues for managing comorbid conditions—such as the coexistence of atopic dermatitis and asthma or chronic obstructive pulmonary disease (COPD). Looking forward, therapeutic paradigms may shift from symptom-focused interventions to the restoration of systemic microbiota homeostasis, advancing a comprehensive “skin–lung axis” model of care (Figure 3).

### 3.2. Interactions Between the Skin Microbiota and the Gut Microbiota

The skin and gut represent the body’s largest external and internal barrier systems, respectively, each hosting diverse and dynamic microbial communities [44,45]. Although structurally distinct, accumulating evidence indicates that these microbiota interact through immune, metabolic, and neuroendocrine pathways, forming what is now referred to as the “skin–gut axis” [46,47]. This bidirectional communication plays a pivotal role in maintaining homeostasis, modulating inflammation, and defending against pathogens.

The gut microbiota contributes to skin health by producing bioactive signaling molecules, such as SCFAs (e.g., butyrate and propionate) and tryptophan metabolites, which are known to regulate immune tolerance and inflammatory processes [48]. It also shapes systemic immune responses by modulating Tregs and T helper 17 (Th17) cells [49], thereby influencing the pathogenesis of various skin diseases. Conversely, skin injury or chronic dermatoses can release inflammatory cytokines, including IL-1β and tumor necrosis factor-alpha (TNF-α) [50], which may impair intestinal barrier integrity and disrupt the gut microbial composition. For instance, studies in murine models have shown that skin barrier damage can upregulate colonic REG3 expression [51], altering gut ecology, while UVB-induced skin aging has been linked to shifts in the relative abundance of key microbial phyla such as Firmicutes and Bacteroidetes [52].

These interconnections suggest that targeting the gut microbiota represents a promising therapeutic approach for managing skin disorders. Interventions such as probiotics (e.g., *Bifidobacterium* and *Lactobacillus*), prebiotics (e.g., fructooligosaccharides), and dietary modifications can enhance gut barrier function, attenuate systemic inflammation, and improve certain conditions, including eczema, acne, and psoriasis [53]. Complementary strategies include preserving the skin’s barrier integrity, minimizing exposure to external irritants, and avoiding over-cleansing. Notably, ultraviolet-based therapies such as narrowband UVB (NB-UVB) have been employed, not only for their dermatologic effects but also for their capacity to indirectly modulate the gut microbiota in patients with psoriasis.

The clinical integration of the “skin–gut axis” concept is gaining traction, offering comprehensive strategies for managing inflammatory skin conditions such as atopic dermatitis, acne, psoriasis, and rosacea. Oral probiotic supplementation has demonstrated benefits for children with eczema, while gut microbiota profiling now supports personalized dietary interventions and microbiota restoration therapies in psoriasis patients. In high-risk groups, such as immunocompromised individuals, modulating the gut microbiota may reduce colonization by opportunistic pathogens and may subsequently lower the incidence of secondary skin infections. However, careful risk assessment is essential: while low-risk individuals may be suitable candidates for synbiotic therapy, its use is strictly contraindicated in vulnerable populations such as infants with severe combined immunodeficiency (SCID) issues [54].

### 3.3. Interactions Between the Lung Microbiota and the Gut Microbiota

Although anatomically distinct, the lungs and intestines function in concert to sustain a complex and dynamically regulated microbial ecosystem. The concept of the gut–lung axis has gained increasing empirical support, emphasizing the critical role of the gut microbiota in maintaining pulmonary immune homeostasis and modulating susceptibility to respiratory diseases. Furthermore, the immune status and microbial composition of the lungs can reciprocally influence gastrointestinal physiology, forming a bidirectional regulatory network that is mediated by microbiota-derived signals [55].

Gut microbes affect lung immunity through several mechanisms, including the production of SCFAs, bile acid metabolites, microbial toxins, and the activation of pattern recognition receptors such as Toll-like receptors (TLRs) [56]. Disruption of the intestinal microbiota, whether through dysbiosis or inflammation, can compromise gut barrier integrity. This facilitates the systemic translocation of pro-inflammatory molecules, alters immune cell trafficking [57,58], and triggers downstream immune responses in the lungs. For example, in models of pulmonary arterial hypertension, an elevated *Firmicutes*-to-*Bacteroidetes* ratio serves as a robust marker of dysbiosis [59]. In lung cancer patients, the gut microbiota shows stage-specific alterations, most notably a reduction in butyrate-producing genera such as *Roseburia* and *Lachnospira* [60]. Similarly, individuals with COVID-19 exhibit marked reductions in gut microbiota diversity, including the depletion of beneficial species such as *Faecalibacterium prausnitzii* and *Eubacterium rectale*, alongside the enrichment of opportunistic pathogens like *Clostridium hathewayi* and *Ruminococcus* spp., underscoring the gut microbiota’s immunoregulatory influence on respiratory pathology [61,62,63].

Conversely, the lung and oral microbiota can influence the gut microbiota through both mechanical and physiological routes [64]. Pulmonary secretions expelled via coughing or mucociliary clearance may be swallowed, introducing respiratory microbiota into the gastrointestinal tract. Opportunistic oral microbiota such as *Porphyromonas gingivalis* (*P. gingivalis*) and *Fusobacterium nucleatum* can migrate to and colonize the gut under permissive conditions, provoking local inflammation [65,66]. Multiple studies have reported a bidirectional relationship between periodontitis and inflammatory bowel disease (IBD) [67,68,69,70], whereby individuals with one condition are at an increased risk of developing the other. This may be mediated by the transmucosal migration of pathogens such as *Klebsiella pneumoniae* and *P. gingivalis*, creating a microbial “bridge” between mucosal surfaces [71]. Experimental models further demonstrate that *Mycoplasma pneumoniae* infection can exacerbate both respiratory and gastrointestinal symptoms, likely via lung–gut microbial crosstalk [72].

Within this framework, the therapeutic modulation of the gut microbiota is emerging as a strategy to enhance respiratory health. For instance, isovaleric acid—a gut-derived metabolite—has been shown to mitigate H9N2 influenza virus-induced pulmonary infection, illustrating the antiviral potential of microbial metabolites [73]. Research by Alashkar Alhamwe et al. indicates that intranasal administration of *Acinetobacter lwoffii* in asthmatic mice can remodel the gut microbiota and elevate IL-6 levels in both bronchoalveolar lavage fluid (BALF) and lung tissue. This IL-6 upregulation contributes to symptom alleviation by reducing pulmonary inflammation through immunomodulatory mechanisms [74]. Similarly, FMT has improved treatment outcomes in murine models of *Klebsiella pneumoniae*-induced pneumonia [75], reinforcing the potential of gut-targeted therapies in respiratory infections. Additional insights come from studies demonstrating that *P. gingivalis*-stimulated CD4^+^ T cells exacerbate dextran sulfate sodium (DSS)-induced colitis by skewing the Th17/Treg balance and expanding pro-inflammatory lymphocyte populations [76,77]. However, co-stimulation with *Lactobacillus rhamnosus GG* reverses this effect by reducing the Th17/Treg ratio and activating the JAK–STAT signaling pathway to suppress inflammation [78].

With advances in metagenomics, metabolomics, and immunomics, future research is well-positioned to dissect the molecular circuits that underlie gut–lung communication in greater detail. Such efforts are expected to yield novel biomarkers and therapeutic targets for the early detection, stratification, and individualized treatment of respiratory diseases. More broadly, integrative therapeutic strategies addressing the gut–lung axis and other inter-organ microbial networks may reshape the prevention of and management paradigms for chronic inflammatory conditions.

## 4. The Immune Crosstalk Mechanism of the Skin–Lung–Gut Axis Microbiota in Allergic Diseases

The regulatory role of the microbiota in various systemic diseases has garnered growing attention. Of particular interest is its involvement in allergic disorders, whereby the interconnected microbial networks of the skin, lungs, and gut—collectively referred to as the “skin–lung–gut axis”—have become a central focus of current research [79]. This concept challenges the traditional notion of organ autonomy, revealing intricate systemic interactions among the epithelial barriers, mucosal immune responses, and resident microbial communities. Clinically, compromised skin barrier function during infancy—a condition that is frequently observed in atopic dermatitis—is often associated with gut microbiota dysbiosis and an increased risk of subsequent respiratory allergies, a phenomenon commonly termed “allergy progression” [80]. This trajectory implies the existence of dynamic, microbiota-mediated immune pathways linking the skin, lungs, and gut, with microbial taxa and their metabolic products acting as crucial mediators.

Immune crosstalk within the skin–lung–gut axis operates through multiple layers of regulation. A key driver is the systemic impact of microbial dysbiosis and its associated metabolites. Studies consistently demonstrate that individuals with atopic dermatitis exhibit markedly reduced gut microbial diversity, particularly with the significant depletion of beneficial genera such as *Bifidobacterium* and *Lactobacillus* [81,82]. This microbial imbalance impairs the production of SCFAs—most notably butyrate, acetate, and propionate—which are essential immunomodulatory molecules that promote Tregs induction and suppress Type 2 helper T cell-driven allergic responses [83]. A deficiency in SCFAs compromises systemic immune regulation, enhancing inflammatory processes and increasing susceptibility to allergic manifestations.

Another central mechanism involves chemokine-mediated immune cell migration across organ systems, establishing immunological continuity between otherwise distant tissues. In murine models of food allergy, intestinal allergen exposure prompts the epithelial cells to secrete chemokines such as CCL20 [84], facilitating eosinophil trafficking to the lungs and promoting a gut–lung inflammatory axis. This underscores the concept of cross-organ immune coordination. Moreover, bidirectional regulation between the microbiota and immune system is further supported by evidence that pulmonary exposure to lipopolysaccharide (LPS) significantly alters the gut microbiota composition of mice [85]. Conversely, the rectal administration of LPS in microbiota-depleted mice restores allergen-specific pulmonary immune responses [86], highlighting the reciprocal influence between gut and lung immune environments via microbial and molecular signals.

A further regulatory layer is provided by the systemic effects of microbial metabolites, which exert influence through circulatory dissemination. For instance, oral administration of ginsenoside F2 has been shown to alleviate skin inflammation in atopic dermatitis mouse models [87]. This effect is mediated by modulation of the gut microbiota, particularly through the enrichment of *Bacteroides* and *Lactobacillus* species, which enhance SCFA production—especially propionic acid. These metabolites exhibit systemic immunoregulatory activity, restoring immune homeostasis in the skin and demonstrating the therapeutic potential of gut-derived microbial products for distal organs.

Together, these findings delineate a complex, dynamic, and interdependent regulatory network that interlinks the skin, lungs, and gut. Disruption of this network may drive the onset and progression of multi-organ allergic diseases. Therefore, unraveling the mechanisms of immune crosstalk within the skin–lung–gut axis not only advances our understanding of allergic pathophysiology but also lays the foundation for integrated microbiota-based therapeutic strategies aimed at systemic allergy modulation.

## 5. The Role of Common Microecological Regulators in the Treatment of Allergic Diseases

### 5.1. Probiotics

Probiotics have recently garnered increasing interest as a promising therapeutic strategy for allergic diseases. Defined by the World Health Organization as live microorganisms that confer health benefits when administered in adequate amounts, probiotics exert their effects through multiple immune-regulatory and microbiota-modulating mechanisms [88]. They support the growth of beneficial microbial taxa, suppress pathogenic microbiota, and inhibit toxin production, thereby optimizing gut microbial composition and promoting immune homeostasis. In addition, probiotics activate immune cells such as dendritic cells and macrophages, stimulate cytokine production, and help rebalance the Type 1/Type 2 cytokine axis—thereby attenuating the predominance of Type 2 immune responses that are commonly associated with allergic disease [89]. Moreover, they produce SCFAs and other metabolites that reinforce mucosal barrier integrity, inhibit the growth of harmful microbes, and activate host receptors such as G protein-coupled receptor 41 (GPR41) and GPR109A [90,91]. These pathways upregulate anti-inflammatory mediators, including IL-10 and aldehyde dehydrogenase 1A1 (*ALDH1A1*), promote Tregs differentiation, and collectively enhance immune tolerance.

A variety of probiotic strains have demonstrated anti-allergic effects in both preclinical and clinical settings. *Lactobacillus rhamnosus* has been shown to reduce the severity of atopic dermatitis in infants and, when administered during pregnancy, to increase TGF-β levels in breast milk [92]. *Lactobacillus plantarum DPUL-F232* lowers serum IgE and histamine concentrations, thereby alleviating intestinal inflammation in allergic murine models [93]. *Bifidobacterium* supplementation has also proven effective in improving infantile eczema [94]. Mechanistic investigations reveal that *Bifidobacterium strain 35624* enriches CD103^+^ dendritic cells in the intestinal mucosa. In concert with retinoic acid and TGF-β, this facilitates the differentiation of Foxp3^+^ Treg cells, reinforcing mucosal immune tolerance [95]. *L. rhamnosus GG* exhibits strong adhesion to intestinal epithelial cells, promoting robust colonization and forming a physical barrier against luminal allergens [96]. Additionally, *L. paracasei AH2* synthesizes SCFAs such as acetic, propionic, and butyric acids [97], which activate the GPR signaling pathways and further support Treg cell development, particularly under the influence of retinoic acid.

**Figure 4:**
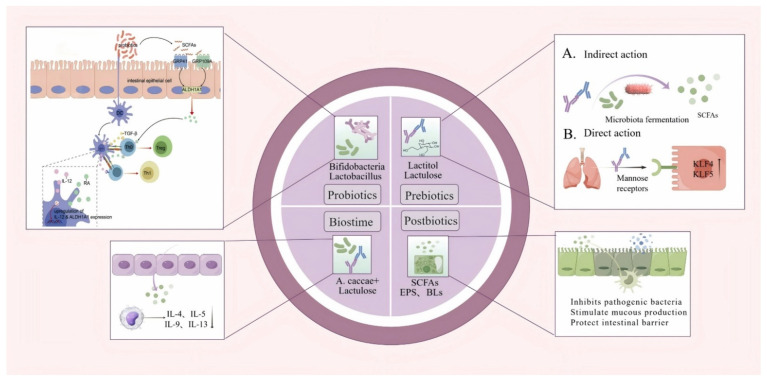
The primary types of microbiota modulators. Composition and primary mechanisms of action of four microbial preparations. This figure was drawn using Figdraw software (https://www.figdraw.com/).

In summary, probiotics offer a multifactorial and biologically plausible approach for the management of allergic diseases. By modulating gut microbiota composition, enhancing mucosal barrier function, restoring immune tolerance, and attenuating systemic inflammation, probiotics present the potential to reduce pharmacological dependency and improve overall quality of life for patients (Figure 4).

### 5.2. Prebiotics

Prebiotics are a class of substances that are selectively utilized by host microbiota to confer health benefits, with their primary function being the regulation of intestinal microecological balance. Unlike probiotics, which involve the direct administration of beneficial microorganisms, prebiotics provide fermentable substrates that promote the proliferation and metabolic activity of specific commensal microbiota, such as *Bifidobacterium* and *Lactobacillus* species [98]. Classic prebiotics include carbohydrate-based compounds—such as lactulose, fructo-oligosaccharides (FOS), galacto-oligosaccharides (GOS), and HMOs—as well as non-carbohydrate compounds like polyphenols [99]. These agents are widely incorporated into functional foods and infant formulas to support gut health and modulate immune function.

Prebiotics exert their effects through both indirect and direct mechanisms. Indirectly, they serve as fermentable substrates for targeted microbial taxa, leading to the production of SCFAs, including acetate, propionate, and butyrate [100]. These SCFAs lower intestinal pH, inhibit colonization by pathogenic microbes, and regulate host immune responses and energy metabolism [101]. They also engage host receptors such as GPR41 and GPR109A, stimulating the expression of immunoregulatory mediators, including IL-10 and *ALDH1A1*, which promote Treg cell differentiation and strengthen systemic immune tolerance [102,103]. Directly, prebiotics can interact with host epithelial cells via receptor-mediated pathways. For instance, mannan-based prebiotics bind to mannose receptors and stimulate bronchial epithelial cell migration and tissue repair [104]. Furthermore, prebiotics can upregulate Krüppel-like factors (KLFs), promote epithelial cell differentiation and proliferation, and enhance the expression of galectin-9. These effects collectively contribute to reduced allergic skin reactions and mast cell degranulation, while also shifting the immune response toward Th1 activity and supporting Treg function to maintain immune equilibrium.

The therapeutic potential of prebiotics for preventing and managing allergic diseases is increasingly supported by empirical evidence. Inulin supplementation, for example, has been shown to significantly increase the abundance of *Bifidobacterium* and *Lactobacillus* while reducing levels of *Clostridium difficile*, thereby preventing the translocation of pathogens from the gut into systemic circulation [105]. In a pivotal randomized controlled trial, Arslanoglu et al. demonstrated that infants fed with a prebiotic oligosaccharide-enriched formula during the first six months of life exhibited a significantly lower incidence of allergic rhinoconjunctivitis and urticaria over a five-year follow-up period, underscoring the critical role of prebiotics in early immune development and allergy prevention [106].

### 5.3. Synbiotics

As research has progressed, the concept of “synbiotics” has emerged—referring to the synergistic combination of probiotics and prebiotics, which interact through complementary mechanisms to enhance host health outcomes. In 2019, the International Scientific Association for Probiotics and Prebiotics (ISAPP) redefined synbiotics as “a mixture comprising live microorganisms and substrates selectively utilized by host microorganisms that confers a health benefit on the host” [107,108]. The central advantage of synbiotics lies in their synergistic mechanism of action: prebiotics serve as selective metabolic substrates that promote the colonization, growth, and functional activity of targeted probiotic strains within the gastrointestinal tract, thereby amplifying the beneficial effects of the probiotics [109]. Compared to the administration of probiotics or prebiotics alone, synbiotics provide more sustained and robust improvements in gut microbial composition, immune homeostasis, and intestinal barrier integrity. A compelling example of synbiotic efficacy was demonstrated by Lauren A. Hesser and colleagues, who evaluated its therapeutic potential in models of allergic disease [110]. The researchers isolated an *Anaerostipes caccae* strain from the feces of healthy infants and combined it with lactulose to formulate a synbiotic. The resulting preparation was then administered to germ-free or microbiota-depleted mice. This synbiotic intervention significantly restored gut microbial diversity, increased cecal butyrate concentrations, and elevated the proportion of CD4^+^CD25^+^ regulatory follicular helper T (Tfr) cells in colonic lymphoid tissue. Additionally, the treatment suppressed IgE-producing cells and attenuated allergic responses to food antigens.

Clinical studies have further reinforced the therapeutic promise of synbiotics in allergic disorders. In one investigation, synbiotic supplementation effectively suppressed Type 2 immune responses induced by grass pollen extract (GPE) in peripheral blood mononuclear cells (PBMCs) derived from allergic patients [111]. Moreover, the synbiotic modulated the immune response in autologous monocyte-derived dendritic cells (MoDCs) and T cell co-culture systems, illustrating its capacity to shape both the local gut ecology and systemic immune function in allergy management.

### 5.4. Postbiotics

Beyond probiotics, prebiotics, and synbiotics, postbiotics have emerged as a promising new category of interventions that are receiving increasing attention. Defined by Tsilingiri et al. as non-viable microbial products or metabolic byproducts that confer health benefits, postbiotics deliver effects that are comparable to those of probiotics without the inclusion of live microorganisms [112]. Broadly, postbiotics encompass two principal components: microbial metabolites and structural elements derived from microorganisms [113]. Microbial metabolites include SCFAs, proteins, vitamins, and other bioactive compounds, with SCFAs being the most extensively studied. These are the primary fermentation products of dietary polysaccharides fermented by the gut microbiota and include acetate, propionate, and butyrate. These SCFAs play key roles in modulating intestinal pH, regulating immune responses, and influencing host energy metabolism. Structural components include microbial lysates (BLs), extracellular polysaccharides, and microbial cell wall fragments such as lipopolysaccharides, all of which exhibit immunomodulatory, anti-inflammatory, and antimicrobial properties [114]. Microbial lysates serve as prototypical postbiotics and consist of antigenic mixtures obtained through the lysis of commonly encountered respiratory pathogens, including *Streptococcus pneumoniae*, *Haemophilus influenzae*, *Moraxella catarrhalis*, *Streptococcus pyogenes*, *Streptococcus viridans*, *Staphylococcus aureus*, *Klebsiella pneumoniae*, and *Klebsiella oxytoca* [115]. A systematic review conducted in 2020 found that the use of BLs as adjunctive therapy significantly reduced the frequency of wheezing and asthma exacerbations in children (*p* < 0.001 for both issues) [116], thereby supporting their clinical efficacy for managing respiratory allergic conditions. Additionally, the review noted the beneficial effects of BLs for reducing episodes of allergic rhinitis and alleviating the symptoms of atopic dermatitis [117].

## 6. Discussion

Microbiota also hold significant therapeutic potential in other disease areas, particularly within innovative strategies aimed at restoring intestinal microbiota homeostasis.

A noteworthy example is FMT, a novel therapeutic intervention designed to reestablish the gut microbial balance. FMT has shown promising outcomes in conditions such as IBD and Parkinson’s disease (PD) [118,119]. In a DSS-induced ulcerative colitis (UC) animal model, FMT significantly decreased the disease activity index (DAI), reduced inflammatory cytokine levels and oxidative stress, and ameliorated histopathological damage [120]. Similarly, a clinical study among Parkinson’s patients with comorbid constipation reported not only marked improvements in gastrointestinal symptoms and motor functions, such as leg tremors, but also showed the significant restoration of gut microbiota composition [121]. Beyond FMT, advancements in probiotic delivery systems and synthetic biology have broadened the therapeutic horizon of microbiota-based interventions. One innovative approach is a cell–probiotic combination strategy, wherein macrophages are utilized as vehicles to deliver encapsulated *Escherichia coli Nissle 1917* (*ECN*) [122]. This method enables targeted delivery, reinforces the mucosal barrier, and mitigates inflammation. The macrophage membrane actively absorbs pro-inflammatory cytokines such as TNF-α and IL-1β, while the encapsulated gel matrix improves probiotic viability and colonization efficiency.

In parallel, engineered probiotics are emerging at the intersection of synthetic biology and materials science. Techniques such as *CRISPR/dCas9*-mediated gene modulation allow for the precise targeting of host inflammatory pathways, enhancing the anti-inflammatory capabilities of probiotics [123]. Meanwhile, chemical engineering approaches—such as the use of biocompatible Eudragit L100-55 enteric coatings—have been employed to improve bioavailability [124]. For instance, Eudragit-encapsulated ECN, which was engineered to express interleukin-2 (ECN-IL2), demonstrated robust intestinal retention, significantly activated Tregs, and promoted epithelial barrier repair in DSS-induced IBD models.

Moreover, targeted screening platforms for assessing the heterogeneity of influenza virus infections have identified specific gut microbial strains and metabolites with strong antiviral properties. Although these emerging microbiota-based therapies have not yet been fully evaluated in allergic disease models, their established roles in immune modulation, inflammation reduction, and microbial ecosystem restructuring suggest meaningful translational potential in allergic contexts.

Another promising avenue involves the interplay between tryptophan (Trp) metabolism and the gut microbiota [125]. Previous studies indicate that Trp and its endogenous metabolites interact closely with intestinal microbes, influencing both local and systemic immunity [126]. Therapeutic strategies leveraging this axis have shown efficacy for treating a range of inflammatory and infectious diseases. Consequently, a deeper exploration of microbial metabolic products—particularly within the context of the “skin–lung–gut axis”—may open new therapeutic avenues for allergic disorders.

Vaccination remains a cornerstone in the prevention and control of respiratory infections. A large-scale, multicenter, double-blind Phase III clinical trial conducted across 18 countries demonstrated that maternal immunization with the RSVpreF vaccine significantly prevented severe lower respiratory tract infections (LRTIs) caused by respiratory syncytial virus (RSV) in infants [127]. Similarly, the RSVPreF3 OA vaccine substantially reduced the incidence of RSV-related LRTIs and acute respiratory infections (ARIs) among high-risk elderly individuals [128]. Building on these successes, microbiota-derived modulators—including probiotics, prebiotics, synbiotics, and postbiotics—are being explored as next-generation vaccine adjuvants. By harnessing the immunomodulatory capacity of gut microbiota and their metabolites, it may be possible to develop personalized adjuvant systems that enhance vaccine efficacy, particularly in the members of vulnerable populations such as pregnant women, neonates, and immunocompromised older adults [129].

Correcting gut microbiota dysbiosis may, thus, become a cornerstone strategy in precision medicine, optimizing immune responses to both pathogens and vaccines, and paving the way for personalized preventive interventions.

With growing insights into host–microbiota interactions, the “skin–lung–gut axis” has gained traction as a therapeutic target in allergic disease management. This interconnected mucosal immune network is orchestrated via microbial metabolites (e.g., SCFAs), cytokine signaling, and neuroendocrine pathways. Precision modulation through microbiota-targeted therapies may allow for the concurrent restoration of immune balance and microbial homeostasis across multiple organ systems. Both clinical and preclinical studies support this concept. For example, a clinical trial in patients with enteric dermatologic conditions found that the oral administration of *E. coli Nissle* significantly reduced erythematous papules and shifted the gut microbiota toward a more protective configuration [130]. In animal models, *Lactobacillus acidophilus TW01* mitigated PM2.5-induced pulmonary injury and enhanced gut microbial diversity [131]. Similarly, *Lactobacillus rhamnosus GG* has demonstrated its efficacy in managing respiratory infections and cystic fibrosis in pediatric populations [132], reinforcing the viability of targeting the gut–lung axis for immune and allergic disease interventions.

## 7. Conclusions

While selected probiotics have demonstrated clinical benefits, further studies are required to define the optimal strains, dosages, and treatment timelines. The use of unverified probiotics with no documented efficacy should be discouraged [133].

In conclusion, the strategic modulation of the skin–lung–gut axis through tailored delivery systems and functional microbiota engineering offers organ-specific and pathway-specific treatment opportunities. This emerging paradigm enhances therapeutic precision and safety, enabling cross-organ immune regulation for allergic diseases, IBD, and respiratory immune disorders. Future integration of host microbiota profiles, immune phenotypes, and clinical signatures may ultimately enable the realization of fully personalized microbiota-based medicine.

## Data Availability

Not applicable.
